# NMR-based plasma metabolic profiling in patients with unstable angina

**DOI:** 10.22038/IJBMS.2020.39979.9475

**Published:** 2020-03

**Authors:** Mohammad PouralijanAmiri, Maryam Khoshkam, Reza Madadi, Koorosh Kamali, Ghassem Faghanzadeh Ganji, Reza Salek, Ali Ramazani

**Affiliations:** 1 Department of Genetics & Molecular Medicine, Faculty of Medicine, Zanjan University of Medical Sciences, Zanjan, Iran; 2 Chemistry Group, Faculty of Basic Sciences, University of Mohaghegh Ardabili, Ardabil, Iran; 3 Department of Cardiology, Mousavi Hospital, Zanjan University of Medical Sciences, Zanjan, Iran; 4 Zanjan Metabolic Diseases Research Center, Zanjan University of Medical Sciences, Zanjan, Iran; 5 Cardiac Surgery Department, Rohani Hospital, Babol University of Medical Sciences, Babol, Iran; 6 International Agency for Research on Cancer, 150cours Albert Thomas, 69372 Lyon CEDEX 08, Lyon, France; 7 Cancer Gene Therapy Research Center, Zanjan University of Medical Sciences, Zanjan, Iran

**Keywords:** Biomarker, Metabolites, Metabolomics, NMR spectroscopy, Unstable angina

## Abstract

**Objective(s):**

Unstable angina (UA) is a form of the acute coronary syndrome (ACS) that affects more than a third of the population before age 70. Due to the limitations of diagnostic tests, appropriate identification of UA is difficult. In this study, we proceeded to investigate metabolite profiling in UA patients compared with controls to determine potential candidate biomarkers.

**Materials and Methods:**

Ninety-four plasma samples from UA and 32 samples from controls were analyzed based on 1H NMR spectroscopy. The raw data were processed, analyzed, and subjected to partial least squares-discrimination analysis (PLS-DA), a supervised classification method with a good separation of control and UA patients was observed. The most important variables (VIP) ≥1 were selected and submitted to MetaboAnalyst pathway enrichment to identify the most important ones.

**Results:**

We identified 17 disturbed metabolites in UA patients in comparison with the controls. These metabolites are involved in various biochemical pathways such as steroid hormone biosynthesis, aminoacyl-tRNA biosynthesis, and lysine degradation. Some of the metabolites were deoxycorticosterone, 17-hydroxyprogesterone, androstenedione, androstanedione, etiocholanolone, estradiol, 2-hydroxyestradiol, 2-hydroxyestrone, 2-methoxyestradiol, and 2-methoxyestrone. In order to determine test applicability in diagnosing UA, a diagnostic model was further created using the receiver operator characteristic (ROC) curve. The areas under the curve (AUC), sensitivity, specificity, and precision were 0.87, 90%, 65%, and 91%, respectively, for diagnosing of UA.

**Conclusion:**

These metabolites could not only be useful for the diagnosis of UA patients but also provide more information for further deciphering of the biological processes of UA.

## Introduction

Acute coronary syndrome (ACS) is a type of coronary artery disease (CAD) that refers to a range of conditions compatible with acute myocardial ischemia and/or infarction due to a sudden reduction in coronary blood flow (1). ACS is classified into myocardial infarction (MI) and unstable angina (UA). UA is a condition in which the heart does not get enough blood and oxygen supply. This condition may be dangerous and can lead to a heart attack. (2-5). The mortality rate in patients with UA was reported at 4.5% after 30 days and increased by 8.6% within six months (6). Despite recent advances in the treatment of ACS, the disease is still a major cause of death worldwide. Early diagnosis of ischemic complications in the heart coronary artery is essential for assessing the consequences of ACS and evaluating the response to therapeutic interventions. Lack of appropriate biomarkers hampers early detection, evaluation, prevention, and risk management in ACS (7). There are two possible mechanisms for the development of ACS: first, rupture of plaque’s fiber cap, and second, the erosion of plaque in the intima (8). The main problem in clinical practice is that symptoms appear at the end of the disease. In fact, plaque formation is an asymptomatic process that causes silent tissue damage. Consequently, the plaque is ruptured, resulting in atherothrombotic events (9). Although several risk factors, such as diabetes, smoking, hypertension, and hyperlipidemia are involved in the early onset of the disease, the precise molecular mechanism is unknown. Furthermore, cardiac biomarkers such as troponin I and T and CK-MB usually cannot be detected until 3-6 hr after MI. Often, more tissue injury is required for the detection of myocardial injury because CK-MB is much less sensitive than troponin (10). Besides, exercise testing with myocardial perfusion imaging is relatively accurate, but it is costly (11), and slow in terms of speed for an emergency department (12). UA is not recognizable in the early stages, and symptoms alone are not adequate to distinguish between different ACS types (13). Due to the above limitations, finding new biomarkers using non-invasive techniques is an urgent need. Advances in “omic” technologies have advantages such as sensitivity, speed, and robustness for identifying new biomarkers in cardiovascular diseases (CVD) (9). 

One of the new “omic” approaches is the field of metabolomics. To understand the disease, the metabolism provides a “snapshot” of all metabolites in a biological sample such as blood, plasma, serum, urine, and many other samples that may be obtained from the patients or the experimental models (14). Metabolites are described as primitive reporters of disease because their increase in biological specimens are often directly associated with pathogenic mechanisms(15). One of the highly used techniques is the proton NMR (^1^H NMR), which has functions for analysis of urine and plasma specimens. Some of the benefits of NMR include no need for degradation and chemical manipulation of specimens and also providing accurate information from the molecular concentrations and structures of metabolites (16). Contrary to genes and proteins that are regulated epigenetically and by post-translational modifications, respectively, metabolites serve as direct signatures of biochemical activity and hence are more easily associated with phenotypes (17). Metabolic changes due to a particular disease can be detected in biological fluids before clinical symptoms develop and produce useful fingerprints (18). Metabolomics has been served for new biomarker discovery and consequently better diagnosing and characterizing of disease, including cancers (brain, lung, ovarian, and breast), neurological disorders (Alzheimer), inborn errors in metabolism (argininosuccinic aciduria, tyrosinemia type II, homocystinuria, and phenylketonuria), diabetes mellitus, tuberculosis, and others (19, 20). 

Metabolomics will increase our understanding of the pathophysiological processes involved in UA and help us to identify potential novel biomarkers to develop new diagnostic strategies (16, 21). In the past, there have been few studies investigating the metabolomics approach for identifying novel biomarkers in UA patients in comparison with healthy controls (22-25). In this study, we have used ^1^H NMR-based spectrometry metabolic profiling of UA patients to find possible candidate metabolites and to provide useful information about important altered metabolic pathways**. **Our study was the first study carried out in Iran on UA using ^1^H NMR. Also, this is the first study in which obstruction has been measured in all three coronary heart arteries. More importantly, the first study is on which controls had symptoms similar to UA without having any obstruction in all three major coronary arteries in angiography. In some studies based on metabolomics approach about UA and CVD, investigators used healthy controls (22, 24-26) and some other controls such as atherosclerosis patients (23) to compare the metabolic profile between patients and controls. Also, in some of these studies, coronary angiography was not used to select patients and controls and was only applied to the symptoms of the disease and the wave of S-T segment changes (24). 

## Materils and Methods

***Clinical characteristics of patients***

From September 2017 to May 2018, we recruited patients with symptoms of UA who referred to the emergency department at Mousavi Hospital and Rohani Hospital, Zanjan and Babol, Iran, respectively. All patients underwent coronary angiography and the percentage of obstruction was determined in the three main coronary arteries: Left Anterior Descending (LAD), Left Circumflex Artery (LCX), and Right Coronary Artery (RCA). From the patients with UA symptoms who were a candidate for coronary artery bypass grafting (CABG) and percutaneous coronary intervention (PCI), we selected 94 UA patients based on having an obstruction in any of the coronary arteries, and also 32 controls that did not have any obstruction in their coronary arteries. The demographic data for the patients with UA and the controls are shown in Table 1. 

***Ethics, consent, and permissions***

Diagnosis criteria of UA refer to the ‘‘2014 AHA/ACC guideline for the management of patients with Non–ST-Elevation Acute Coronary Syndromes’’. All stages were carried out in accordance with the Helsinki Declaration. The study was ethically approved by the ethics committee of the Zanjan University of Medical Science (ethics approval code, ZUMS.REC.1396.98). Informed consent was taken from all patients before their inclusion in the study.

***Inclusion, exclusion, and rejection criteria***

All selected UA and the controls were diagnosed by physical examination, cardiac biomarkers, and confirmed by coronary angiography. Patients with obstruction over 75% in one of the three main coronary arteries (LAD, LCX, or RCA) were included in the UA group. Also, patients who were accepted as UA patients but in angiography did not have any obstruction in coronary arteries were included in the control group. The exclusion criteria include a history of heart, liver, or metabolic disease, diabetes, rheumatism, cancer, hematopoietic system, pulmonary embolism, autoimmune disorders, severe infectious diseases, trauma, and a recent surgical procedure. Also, patient samples whose files were not available or lacked clinical information were excluded from the study. 

***Sample collection, preparation, and NMR spectroscopy***

Five-milliliter blood samples were taken from patients with UA symptoms and placed in tubes containing EDTA as an anticoagulant and immediately centrifuged (2-6E, Sigma, Germany) at 3500 rpm for 10 min to separate plasma. The plasma samples were stored at -80 ^°^C until NMR analysis. The ^1^H NMR data were acquired at room temperature on a Bruker 400 NMR spectrometer using Carr–Purcell–Meiboom–Gill (CPMG) pulse program (27). In brief, 400 μl of plasma samples were mixed with 100 μl of D_2_O. For calibration of the chemical shift, Tetramethylsilane (TMS) was applied as the internal reference in NMR tubes.

***Pre-processing and Multivariate statistical analysis of*** ^1^ ***H NMR spectra***

**Figure 1.**
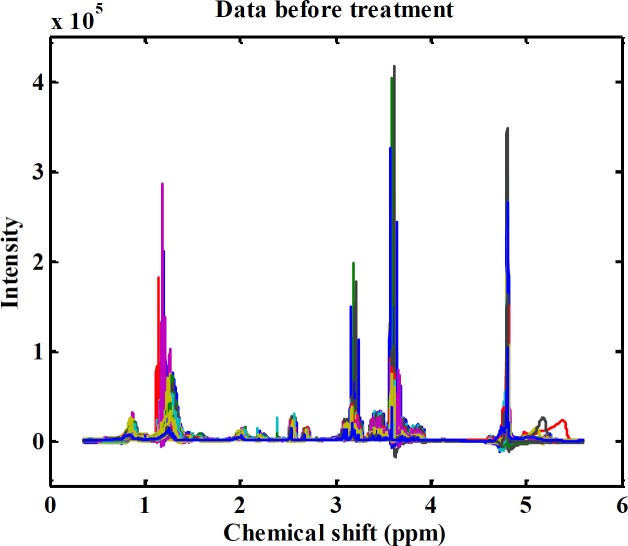
Raw data before any pre-prossecing treatment and analysis

All NMR data in the FID time domain were imported into the MestReNova software package (version 6.0.2-5475) for data processing. The FID data were subjected to Fourier transform to obtain the spectra in the frequency domain. Then data were phased and baseline-corrected and exported as an ASCII file and imported into MATLAB (R2008a; Mathworks, Natick, MA) for further analysis. To eliminate water resonance, the spectral regions from 4.7 to 5 ppm were removed. In the next step, each ^1^H NMR spectrum from plasma was segmented into equal widths (0.003 ppm), corresponding to regions 0–9 ppm. Figure 1 shows the final binned ^1^H NMR spectra of all samples. Then the spectral data were mean-centered and scaled according to the Pareto scaling method (28, 29). 

A home-written program in MATLAB was used for multivariate statistical analysis. After pre-processing of data, principal component analysis (PCA) was applied to data as an unsupervised classification method. Since the separation of groups was not good (data not shown), PLS-DA as a supervised classification method was applied. 

**Figure 2:**
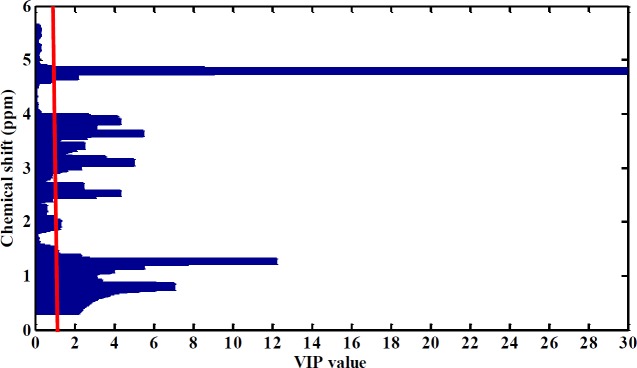
Important variables were selected according to VIP≥1. Vertical red line show the VIP=1. VIP more than 1 selected for analysis

The corresponding loading plot from PLS-DA was obtained, and the important variables were selected according to VIP≥1 (Figure 2). 

**Figure 3:**
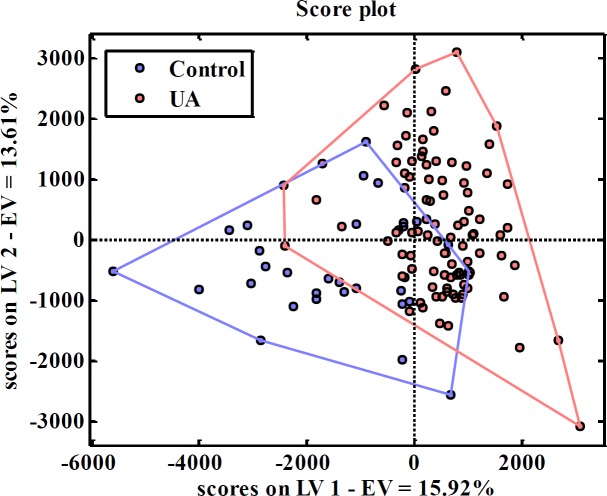
Score plot from applying PLS-DA to data. LV and EV are bbreviations for Latent Variable and Explained Variance, respectively. Red circles indicating UA patients and blue one indicating the controls

Figure 3 shows the score plot of data from PLS-DA. 

The proximity of patient and control profiles can be a good reason for some patients to overlap in the PCA analysis, which probably indicates that the metabolic profile of these patients is close to the metabolic profile of the controls. 

**Figure 4:**
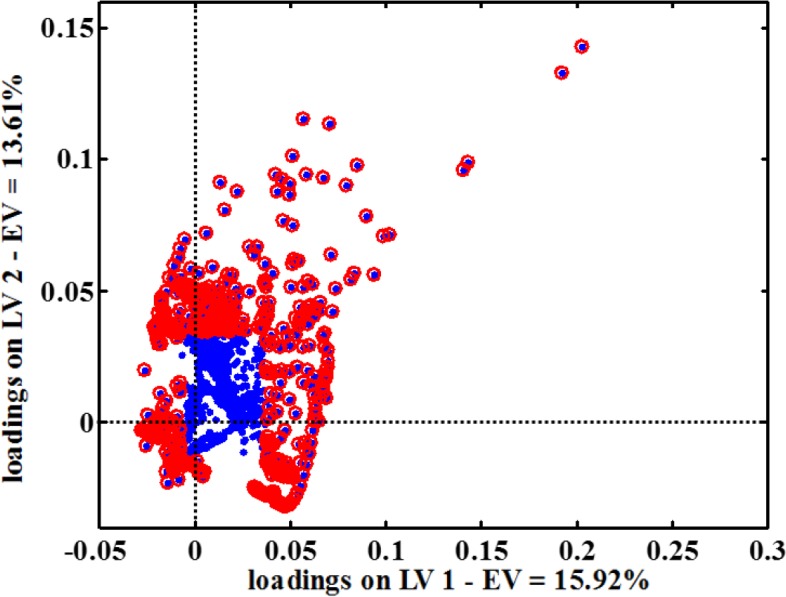
Loading plot from applying PLS-DA to data. The circled points are the points whose corresponding chemical shifts were selected based on their VIP values more than 1

The loading plot is shown in Figure 4, and the important variables are shown in this figure.

***Identification of metabolites and pathways***

Following maximum class separation, the Human Metabolome Database (HMDB) was used to obtain metabolites based on the chemical shifts of class separation (30). Selected chemical shifts that are shown in Figure 4 were entered into HMDB to identify altered metabolites. Then, we uploaded the selected metabolites into MetaboAnalyst 4.0 (31) in order to identify significant altered biochemical pathways based on *P*-values less than or equal to 0.05 (Table 3).

## Results

***Clinical characteristics and demographic information***

Patients were selected based on UA symptoms and lack of cardiac biomarkers and coronary angiography, a golden standard method for detecting atherosclerotic plaques verified the percentage of stenosis in each of LAD, LCX, and RCA. Obstruction over 75% was considered as significant, and patients with at least one blockage were enrolled in the UA group. Patients without any significant obstruction were included in the control group. For patients with UA and control, a data collection form was completed that included clinical characterizations and demographic information of each patient and control. In the next stage, the informed consent form was completed for patients. In our study, NMR spectrometry was carried out for the 204 plasma samples of UA patients and 56 from controls. Some patients in case and control groups were rejected from our study because of incomplete information and having special diseases. Also, diabetic patients in cases and controls were rejected from the study. Therefore, we removed their NMR information from the study. Hence, 94 cases and 32 control samples remained in the study. The detailed demographics and clinical data of the study’s subjects are indicated in Table 1. 

***Metabolite identification***

We applied multivariate analysis to the obtained raw data from NMR spectroscopy to find best chemical shifts. The information about all analysis presented in Figure 2. After pre-processing and multivariate statistical analysis, selected chemical shifts obtained from NMR spectra were uploaded into the Human Metabolome DataBase (HMDB). We selected 17 important metabolites for examining their relationship with CVD, atherosclerosis and also finding their increased or decreased levels. 17 affected metabolites were identified in the 4 significant altered biochemical pathways (Table 2). We considered four significant pathways based on *P*-value less than or about 0.05. 

The important altered biochemical pathways were steroid hormone biosynthesis, aminoacyl-tRNA biosynthesis, lysine degradation, phenylalanine, tyrosine, and tryptophan biosynthesis. The most important metabolites associated with the most important biochemical pathways were deoxycorticosterone, 17-hydroxyprogesterone, androstenedione, androstanedione, etiocholanolone, estradiol, 2-hydroxyestradiol, 2-hydroxyestrone, 2-methoxyestradiol, 2-methoxyestrone, L*-*arginine, L-methionine, L-tryptophan, L-tyrosine, aminoadipic acid, N6-acetyl-L-lysine, L-pipecolic acid. 10 metabolites were altered in the steroid hormone biosynthesis pathway included deoxycorticosterone, 17-hydroxyprogesterone, androstenedione, androstanedione, etiocholanolone, estradiol, 2-hydroxyestradiol, 2-hydroxyestrone, 2-methoxyestradiol, 2-methoxyestrone. 4 metabolites were altered in aminoacyl-tRNA biosynthesis pathway included L*-*arginine, L-methionine, L-tryptophan, L-tyrosine. 3 metabolites were altered in the lysine degradation pathway included aminoadipic acid, N6-acetyl-L-lysine, and L-pipecolic acid. Also, two metabolites L-tyrosine and L-tryptophan were altered in another biochemical pathway called phenylalanine, tyrosine and tryptophan biosynthesis pathway.

Among 17 selected metabolites, 12 metabolites had decreased levels, and 5 metabolites had increased levels in UA patients in comparison to the control group (Table 2). 

**Figure 5:**
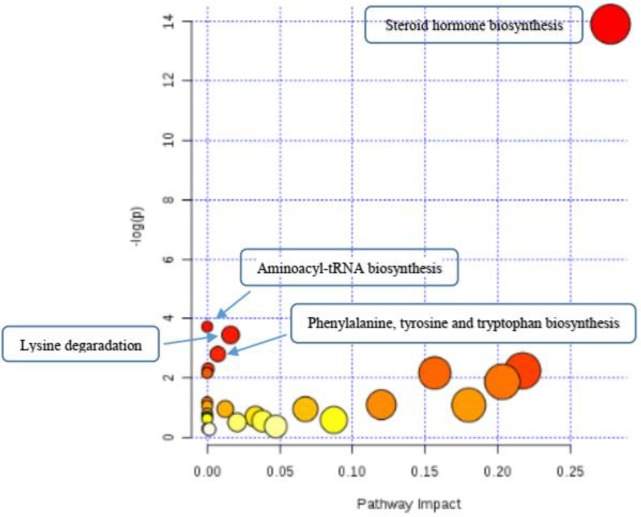
Graphical overview of metabolic pathway topology analysis of unstable angina (UA) metabotypes. The graph was generated using MetaboAnalyst 4.0 web-based software

As shown in Table 2, compared with the controls, UA patients had higher levels of 5 metabolites; deoxycorticosterone, androstenedione, etiocholanolone, 2-hydroxyestradiol and L-methionine and lower levels of 12 metabolites; 17-hydroxyprogesterone, androstanedione, 2-hydroxyestrone, estradiol, 2-methoxyestradiol, 2-methoxyestrone, L*-*arginine, L-tryptophan, L-tyrosine, aminoadipic acid, N6-acetyl-L-lysine, and L-pipecolic acid. The significant altered biochemical pathways are shown in Figure 5, an overview of pathway analysis. As it is observed, the pathway of steroid hormone biosynthesis has high pathway impact value compared with the 3 other biochemical pathways in pathway topology analysis (Table 3).

The significant altered metabolic pathways are shown in Table 3. As noted, we found the important pathways based on *P-values* less than or equal to 0.05. 

**Figure 6:**
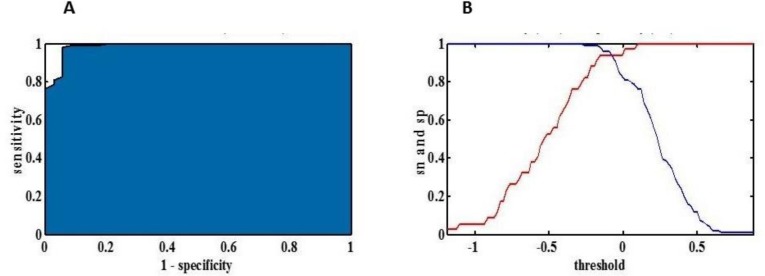
The obtained ROC curve to diagnose unstable angina (UA) patients. A: AUC, B: Specificity (red) and sensitivity (blue)

To validate the PLS-DA model and test and also its applicability in diagnosing UA, the ROC curve analysis was carried out to determine the clinical efficacy of these potential biomarkers. Accordingly, the sensitivity and specificity were determined for the test. The areas under the curve (AUC) for UA were 0.99. The sensitivity, specificity, and precision of the test were 0.87, 90%, 65%, and 91%, respectively, for diagnosing UA (Figure 6).

## Discussion

Because UA can lead to MI, it is important that the disease is diagnosed as soon as symptoms appear, and urgent proceedings are taken to prevent MI. ^1^H NMR metabolomics approach was applied to investigate the novel candidate metabolites in UA patients. We identified some of the disturbed important metabolic pathways in UA patients, which include steroid hormone biosynthesis, aminoacyl-tRNA biosynthesis, lysine degradation, and phenylalanine, tyrosine, and tryptophan biosynthesis pathway. 

The first altered pathway in our study was steroid hormone biosynthesis. The affected metabolites in this pathway were in the group of mineralocorticoid and sex hormones. We identified 10 metabolites in the pathway of steroid hormone biosynthesis including one metabolite from mineralocorticoid group, a class of corticosteroids, and 9 metabolites from the sex hormone group. In mineralocorticoid group, deoxycorticosterone and in sex hormone group 17-hydroxyprogesterone, androstenedione, androstanedione, etiocholanolone, estradiol, 2-hydroxyestradiol, 2-hydroxyestrone, 2-methoxyestradiol, and 2-methoxyestrone were detected compared to the controls. 

Mineralocorticoids are produced in the adrenal cortex and affect electrolyte and fluid balance, such as salt and water. Deoxycorticosterone is a precursor to aldosterone, which is the primary mineralocorticoid. 

Corticosterone, the main glucocorticoid, is produced from deoxycorticosterone involved in the regulation of energy, immune reactions, and stress responses. Glucocorticoids are steroid hormones that have inflammatory and immunosuppressive effects on a wide variety of cells. One of the effects of glucocorticoids is to reduce the expression of pro-inflammatory genes (32). Therefore, this may be due to an increase in the inflammatory response, which is one of the predisposing factors for atherosclerotic plaque formation and rupture. Among these steroid hormones, aldosterone has some major deleterious effects on the cardiovascular system, including endothelial dysfunction and myocardial necrosis (33).

Aldosterone binds to mineralocorticoid receptors (MR) and regulates gene expression involved in sodium retention, potassium secretion, and water reabsorption, all of these actions may result in increased blood pressure. Moreover, MR is expressed in a variety of other tissues, and its activation could lead to tissue injury. Indeed, it has been shown that MR activation in the cardiovascular system promotes hypertension, fibrosis, and inflammation. Confirming this issue, the use of MR antagonists is helpful for patients with heart failure (HF) and prevents mortality and morbidity (34). 

In addition, an increasing number of clinical and experimental evidence suggests that MR is involved in a wide range of diseases, especially CVDs. MR activation is involved under different pathophysiologic conditions of the cardiovascular system, such as high blood pressure, HF, and MI (35). Several mechanisms including oxidative stress, inflammation, and fibrosis show the pathophysiological role of MR in the cardiovascular system (34). Furthermore, it has recently been shown that the proatherogenic effects of aldosterone are mediated by an increase in expression of ICAM-1 induced by endothelial MR activation in an atherosclerotic mouse model (36). Some studies done using animal models show the beneficial effects of MR antagonists on the endothelial dysfunction related to MI (37). 

The other metabolite in the pathway of steroid hormone biosynthesis were sex hormones. Some of the studies mentioned sex steroids have immunomodulating actions, but their roles in inflammation are complex. Understanding the interplay of atherosclerosis and sex steroid hormones and their receptors on the vessel wall has great importance for clinical practice. Atherosclerosis is caused by a chronic inflammatory condition in the vessel wall, which leads to vasoconstriction or obstruction and is associated with vascular dysfunction (38). Other important considerations in the gender differences observed in CVD between men and women are related to a number of biological agents that affect the type of plaque build-up and vascular response to plaque (39). Compared to men or women after menopause, there is a relatively lower CVD in women before menopause (38). Supporting evidence is limited on low levels of testosterone and its metabolites such as dihydrotestosterone (DHT) and estradiol in the genesis of atherosclerosis and cardiovascular disease in older men. Observational studies link lower T levels with carotid atherosclerosis, aortic and peripheral vascular disease, and cardiovascular events and deaths (40).

One of the affected pathways in UA patients was the aminoacyl-tRNA biosynthesis pathway, which consists of four metabolites L*-*arginine, L-methionine, L-tryptophan, and L-tyrosine. Aminoacyl-tRNA synthetases include an old family of enzymes that harbor all cells and exist in three major kingdoms of life. Aminoacyl-tRNA synthetases are necessary and ubiquitous ‘house-keeping’ enzymes responsible for charging amino acids to their cognate tRNAs and giving the substrates to synthesize proteins. They perform the esterification reactions that connect amino acids with their tRNAs bearing the correct anticodon to protect the perfect transfer of information directed by the genetic code. Generally, the aminoacylation reaction includes a two-step process: first activation of amino acids by ATP and creating an intermediate aminoacyl adenylate, and then transfer to the 3′-end of tRNA to form the aminoacyl-tRNA end-product. Recent studies have shown several roles for aminoacyl-tRNA synthetases in diseases and their application as pharmacological targets and therapeutic reagents. In addition to the canonical functions of aminoacyl-tRNA synthetases, including aminoacylation and editing, they have the noncanonical activities that are unrelated to aminoacylation, consisting of translation control, transcription regulation, signal transduction, immune cell migration, endothelial cell detachment, stimulation of endothelial cell migration, angiogenesis, inflammation, and tumorigenesis (41, 42). Charcot–Marie–Tooth disease is an inheritable human disease caused by mutations in cytoplasmic aminoacyl-tRNA synthetases. Diseases related to the heart muscle such as cardiomyopathies caused by the deterioration of the myocardium function result in a mutation in aminoacyl-tRNA synthetases (42). In a study aminoacyl-tRNA biosynthesis was one of the altered pathways in UA patients (22). In this study, 3 altered metabolites L*-*arginine, L-tryptophan, and L-tyrosine decreased in UA patients, but L-methionine increased.

L-arginine is the only substrate of NO production that affects the cardiovascular system. Many studies have clearly shown a beneficial effect of L-arginine on endothelium hypofunction and thus reduced NO synthesis. Additionally, experiments on animals have been performed and *in vitro* data also indicate that L-arginine may have antiaggregatory, anticoagulant, and profibrinolytic effects (43). -Arginine has antioxidant and antiapoptotic effects and increases the relaxation in smooth muscle cells. It also inhibits the expression of adhesion molecules, chemotactic peptides, platelet aggregation, and decreases endothelin-1 expression (44). Production stimulation of endogenous nitric oxide could inhibit atherogenesis, and therefore may be beneficial in patients with risk factors for atherosclerosis (45). In our study, the -Arginine level was lower compared with the controls. L-methionine as a substrate for cysteine, taurine, S-adenosyl methionine, and glutathione, plays a critical role in the metabolism and health of many species. Methionine is also an important part of angiogenesis. In our study, L-methionine level was higher compared with the controls. It has been shown that high level of methionine can be atherogenic in susceptible mice and causes atherosclerosis and CVD (46). Also, it was shown that a high intake of methionine could increase the risk of acute coronary events in middle-aged men (47). High doses of methionine can lead to an acute increase in plasma homocysteine levels, which can be a sign of the susceptibility to CVD (48). It is known that homocysteine, by harmful effects on cardiovascular endothelium and smooth muscle cells, can mediate cardiovascular problems with alterations in subclinical arterial structure and function (49). 

L-tryptophan is an essential amino acid and a major component of protein synthesis in humans and animals. Research has displayed that the -kynurenine pathway has a key role related to the pathological regulation in both innate and adaptive immune systems (50). The activity of indoleamine 2,3-dioxygenase, an enzyme in the kynurenine pathway, indicated a significant association with atherosclerosis risk factors such as age, LDL cholesterol, and BMI into the female population (51). The increased degradation of tryptophan due to the production of pro-inflammatory cytokines produced by monocytes leads to increased kynurenine and inflammation. The increase of kynurenine/tryptophan ratio increases risk of cardiovascular events (52, 53). Furthermore, increase in expression of indoleamine 2,3-dioxygenase was identified in the macrophages in the atherosclerotic plaques in human (54), and also a study illustrated that low levels of tryptophan in plasma and high levels of kynurenine to tryptophan ratio are features of individuals that suffer from coronary heart disease (55). L-tyrosine or 4-hydroxyphenylalanine is a non-essential amino acid with a polar side group. There is evidence that inflammation and immune activation also damage the conversion of phenylalanine to tyrosine. A relationship has been demonstrated in patients suffering from sepsis, cancer, or HIV-1 infection and also in the healthy elderly that have high levels of phenylalanine and as result, phenylalanine to tyrosine ratio (56). It has been described that phenylalanine and Phe/Tyr ratio are associated with some markers of immune activation such as neopterin (57, 58). In a study using a small group of patients, the same associations were seen in patients suffering from CAD (56). The pathway of lysine degradation was another pathway that changed in this study in which three metabolites, aminoadipic acid, N6-acetyl-L-lysine, and L-pipecolic acid were altered. The levels of these metabolites in UA were decreased compared with the controls. 

Aminoadipic acid is an intermediate metabolite in the degradation of lysine and saccharopine. Lysine catabolism occurs through one of several pathways, the most common of which is the saccharopine pathway. Aminoadipic acid may be used as a precursor to the downstream enzyme for tryptophan metabolism. It has also been shown that aminoadipic acid inhibits the production of kynurenic acid (59). As a result, the level reduction of aminoadipic acid may be related to an increase in the level of kynurenine and in turn, with inflammation and atherosclerosis.

 It is known that aminoadipic acid, as an atherogen, is produced by oxidation of lysine in proteins into the atherosclerotic plaques at high levels (60). The role of aminoadipic acid as a marker was verified in oxidative stress (61). Some studies have shown that aminoadipic acid is increased in prostate biopsies obtained from patients with prostate cancer (62). Therefore, in some of the circumstances, aminoadipic acid can have a role as an atherogen and a metabotoxin. An atherogen is a compound that leads to atherosclerosis and CVD. A metabotoxin is an endogenous metabolite that causes adverse effects on human health at chronically high levels. In our study, the reduction of aminoadipic acid can lead to reduction of lysine biosynthesis and consequently may decrease collagen synthesis within the plaque. This event may cause an unstable plaque. L-pipecolic acid is a normal metabolite present in human blood. This metabolite accumulates in body fluids of infants with some of the genetic peroxisomal disorders, such as Zellweger syndrome, neonatal adrenoleukodystrophy, and infantile Refsum disease. The levels of L-pipecolic acid are also raised in patients with chronic liver diseases. L-pipecolic acid is the substrate of delta1-piperidine-2-carboxylate reductase in the pathway of lysine degradation (63-65). 

**Table 1:** Demographic information and clinical characteristics of unstable angina (UA) patients and controls

**Variables**			**Group**		**P-value**
			**UA Patients (n=94)**	**Control (n=32)**	
**Age (Mean± SD)**			64.07 ± 10.5	54.13 ± 11.7	< 0.001
**Gender**		**Male **	54 (57.4 %)	14 (43.8 %)	0.179
		**Female**	40 (42.6 %)	18 (56.3 %)	
**BMI**			26.44 ± 3.9	28.56±4.9	0.023
**Smokers**			9 (12.3 %)	5 (15.6 %)	0.756
**Ex-smokers**			3 (4.1 %)	4 (12.5 %)	0.196
**Hypertension**			44 (60.3 %)	15 (46.9 %)	0.203
**Hyperlipidemia**			47 (64.4 %)	17 (53.1 %)	0.276
**Overweight**			41 (60.3 %)	24 (77.4 %)	0.096
**Triglyceride (mg/dl)**			136.67±58.1	113.13±41.5	0.337
**Total cholesterol (mg/dl)**			182.25±59.5	215.88±99.6	0.355
**HDL(mg/dl)**			45.50±10.6	43.50±8.7	0.664
**LDL(mg/dl)**			101.91±47.1	133.86±83.8	0.313
**Ejection fraction (%)**			48.20±8.3	49.29±11.0	0.635
**Mean LAD stenosis (%)**			91.45±7.2	N/A	_
**Mean LCX stenosis (%)**			67.27±36.8	N/A	_
**Mean RCA stenosis (%)**			68.97±39.2	N/A	_
**Stenosis count**	**Single Stenosis **		6 (8.2 %)	N/A	_
	**Double Stenosis**		16 (21.9 %)	N/A	_
	**Triple Stenosis**		51 (69.9 %)	N/A	_

BMI: Body mass index; HDL: High density lipoprotein; LDL: Low density lipoprotein; N/A: Not applicable; LAD: Left anterior descending; LCX: Left circumflex artery; RCA: Right coronary artery

**Table 2:** Selected important metabolites obtained from unstable angina (UA) patients

**No.**	**Metabolites**	**HMDB ID**	**Pathway**	Change*
1	Deoxycorticosterone	HMDB0000016	Steroid hormone biosynthesis	↑
2	17-Hydroxyprogesterone	HMDB0000374	Steroid hormone biosynthesis	↓
3	Androstenedione	HMDB0000053	Steroid hormone biosynthesis	↑
4	Androstanedione	HMDB0000899	Steroid hormone biosynthesis	↓
5	Etiocholanolone	HMDB0000490	Steroid hormone biosynthesis	↑
6	Estradiol	HMDB0000151	Steroid hormone biosynthesis	↓
7	2-Hydroxyestradiol	HMDB0000338	Steroid hormone biosynthesis	↑
8	2-Hydroxyestrone	HMDB0000343	Steroid hormone biosynthesis	↓
9	2-Methoxyestradiol	HMDB0000405	Steroid hormone biosynthesis	↓
10	2-Methoxyestrone	HMDB0000010	Steroid hormone biosynthesis	↓
11	L-Arginine	HMDB0000517	Aminoacyl-tRNA biosynthesis	↓
12	L-Methionine	HMDB0000696	Aminoacyl-tRNA biosynthesis	↑
13	L-Tryptophan	HMDB0000929	Aminoacyl-tRNA biosynthesis	↓
14	L-Tyrosine	HMDB0000866	Aminoacyl-tRNA biosynthesis	↓
15	Aminoadipic acid	HMDB0062715	Lysine degradation	↓
16	N6-Acetyl-L-lysine	HMDB0033891	Lysine degradation	↓
17	L-Pipecolic acid	HMDB0000716	Lysine degradation	↓

**Table 3:** The altered metabolic pathways in unstable angina (UA) patients in comparison to controls

**No**	**Pathway**	***P-value***	**-log(p)**	**Holm p**	**FDR**	**Impact**
1	Steroid hormone biosynthesis	9.1778E-7	13.901	7.3422E-5	7.3422E-5	0.27779
2	Aminoacyl-tRNA biosynthesis	0.024081	3.7264	1.0	0.84683	0.0
3	Lysine degredation	0.031756	3.4497	1.0	0.84683	0.01613
4	Phenylalanine, tyrosine and tryptophan biosynthesis	0.060425	2.8064	1.0	1.0	0.00738

## Conclusion

We conducted the first study on the metabolome profile of UA patients in the Iranian population based on NMR-based spectroscopy. The patients with diabetes were excluded from our research in order to eliminate interferences. In our study, some of the altered pathways and metabolites in the UA patients were reported for the first time. We identified two new important disturbed pathways including steroid hormone biosynthesis, phenylalanine, tyrosine, and tryptophan biosynthesis in UA patients. Also, we have reported the aminoacyl-tRNA biosynthesis pathway and lysine degradation that agree with a previous study about UA patients (22). The identified metabolites include 17 new metabolites that have been listed in Table 2. Approximately, most of the identified metabolites were new, and they have not been reported in articles about ACS metabolomics. These results need to be confirmed by other methods for use in clinics, but this finding may provide for a better diagnosis of UA in the future to predict heart attacks in patients.
